# Symptom impact and safety of ketogenic therapy in adults with anorexia nervosa: a feasibility trial

**DOI:** 10.1038/s43856-026-01644-0

**Published:** 2026-06-03

**Authors:** Guido K. W. Frank, Barbara Scolnick, Caroline Beckwith, Megan E. Shott, Shannon Kilty, Skylar Swindle, Michael Lutter, Jong M. Rho

**Affiliations:** 1 University of California San Diego, Department of Psychiatry, San Diego, CA USA; 2 Rady Children’s Health San Diego, San Diego, CA USA; 3 Private Practice, Waban, MA USA; 4 Bronx, NY, USA; 5 Precision Psychiatry, Plano, TX USA; 6 Yale University, New Haven, CT USA

**Keywords:** Phase II trials, Anxiety

## Abstract

**Background:**

Anorexia nervosa (AN) is a severe psychiatric disorder characterized by food restriction and significantly low body weight. Even after weight restoration, core symptoms such as body dissatisfaction, intense fear of eating, and preoccupation with body shape and weight often persist, contributing to a high risk of relapse.

**Methods:**

This study evaluated the feasibility and safety of a weight-maintaining ketogenic dietary therapy administered over 14 weeks in adults with anorexia nervosa who were either mildly underweight or weight-restored. The study also examined whether ketogenic therapy could reduce eating disorder symptoms. Twenty-two individuals participated, and eighteen (82%) completed the trial.

**Results:**

Repeated-measures MANCOVA (intent-to-treat; comorbidity and medication included in the model) reveals significant overall treatment effects (Wilk’s λ = 0.165, F = 2.383, *p* < 0.001), and symptom score decreases over time on the Eating Disorder Examination Questionnaire (EDE-Q) subscales Restraint, Eating Concern, Shape Concern, and Weight Concern, as well as for depression scores. At study completion, 72% of participants show EDE-Q and depression scores within the normal range. At three-month follow-up, 39% of completers continue ketogenic dietary therapy, of whom 28% show an increase in EDE-Q Global scores, while among those not maintaining ketogenic therapy, 64% present with an elevation in EDE-Q scores. Ketogenic dietary therapy does not precipitate worsening of symptoms or clinically significant weight loss.

**Conclusions:**

The ketogenic dietary therapy is well-tolerated and demonstrates potential efficacy in reducing core symptoms of AN among adults who are mildly underweight or weight-restored. These findings support the further investigation of ketogenic dietary therapy as a potential intervention for this population. ClinicalTrials.gov ID: NCT06000774; Registration Date August 14, 2023.

## Plain language summary

Anorexia nervosa is a serious mental health condition marked by food restriction and low body weight. Even after weight restoration, many individuals continue to struggle with intense fears about eating, dissatisfaction with their body, and ongoing worries about weight and shape. This study explored whether a special, fat-based diet could be a safe and effective treatment for adults with anorexia nervosa. Participants tolerated the diet well, with no evidence of worsening of symptoms. Instead, participants showed significant improvements in eating disorder symptoms, including reduced dietary restraint, less concern about food and body shape, and improved mood. By the end of the study, nearly three-quarters of participants had scores within the normal range for both eating disorder and depression symptoms. These findings suggest that a ketogenic diet may be a safe and promising approach to help reduce core symptoms of anorexia nervosa.

## Abstract (web summary)

Frank et al. evaluate the safety and clinical effects of a weight-maintaining ketogenic therapy in adults with anorexia nervosa who were either mildly underweight or weight-restored. The ketogenic therapy is well tolerated and associated with significant improvements in both eating disorder-specific and general psychopathology.

## Introduction

Anorexia nervosa (AN) is a severe psychiatric disorder marked by extreme food restriction and significant weight loss, fear of gaining weight, and a distorted body image^1^. It is a chronic condition with a high risk of relapse, substantial disease burden, and considerable treatment costs^2–6^. Even after weight restoration, individuals with AN frequently continue to experience intense fears of eating and weight gain, and body dissatisfaction, indicating that weight restoration is not sufficient for psychological recovery^2,7–9^. These symptoms can be as severe—or even more pronounced—than during the underweight phase, increasing the risk of relapse^10,11^.

The strong drive for weight loss and self-starvation in AN remains perplexing and has often been likened to compulsive or addictive behaviors^12^. More recent theories suggest that the underlying mechanisms contributing to AN may involve metabolic abnormalities, leading some to propose that AN is a “metabolic disorder of psychological origin”^13,14^. Several studies have identified metabolic irregularities in AN, including signs of increased oxidative stress^13–16^. Recent genetic research indicates that metabolic traits that affect glucose and lipid metabolism may contribute to the risk of developing AN^17^. Mitochondria play a central role in brain energy metabolism, where mitochondrial and nuclear DNA interact and remain susceptible to environmental influences with implications for the development of psychiatric disorders^18–20^. A key component of healthy mitochondrial function is the amino acid derivative carnitine, which supports fatty acid oxidation, protects cellular membranes, and modulates both ketogenesis and gluconeogenesis^21^. A neurobiological model that centered on energy homeostasis proposed that heightened anxiety and stress—commonly observed in AN—play central roles in the development and maintenance of the disorder^22,23^. According to that model, these psychological factors disrupt the balance of glucose distribution between the brain and body, alter the function of the hypothalamus-pituitary-adrenal (HPA) axis, and activate the body’s stress response, leading to an increased demand for glucose by the brain, potentially depleting the body’s energy reserves^22–25^. However, while stress has been linked to increased glucose demand in the brain, some evidence suggests that, in certain individuals, stress may reduce the brain’s ability to utilize glucose^26–28^. More recently, supported by computational models, it has been hypothesized that altered or inefficient brain glucose metabolism is a core feature of several psychiatric disorders—an insight that may also be relevant to the pathophysiology of AN^29–32^.

Emerging research increasingly links nutrition to mental health^33^, and brain metabolic alterations, perhaps relating to mitochondria, have been hypothesized to have a critical role in psychiatric disorders^31,32^. Genetic disorders that affect metabolism have, on the other hand, been associated with psychiatric conditions, supporting the link between altered metabolism and psychiatric pathophysiology^34^. Psychiatric conditions have also been associated with elevated inflammatory markers and markers for oxidative stress^35–37^, and nutrition plays an important role in activation of the immune system^38,39^. It is therefore possible that macronutrient intake and a disturbance in metabolism could also contribute to the pathophysiology of AN.

Individuals with AN often try numerous dietary regimens, particularly those that severely restrict fat intake; however, these behaviors fail to alleviate the core fear of weight gain^40–43^. Current treatments for AN prioritize weight restoration through structured meal plans aimed at achieving target weights^44^. Yet, key symptoms—such as the intense fear of gaining weight and distorted body image—often persist, contributing to high rates of suicidality and relapse^45^. Thus, neither restrictive eating nor weight restoration alone resolves the profound emotional distress related to body shape and weight. Co-author CB, the peer counselor in this study, experienced AN for fifteen years and by the age of 29 years had been to multiple treatment centers. While she was weight normalized, she continued to have the typical cognitive-emotional and body image symptoms that characterize AN. Co-author BS started her on a dietary ketosis regimen, and after 3 months on KT, she had markedly improved and has been in full recovery since^46^. That outcome was supported by a case series of five weight-normalized individuals with AN, who were on KT for between 4 and 8 weeks, prior to receiving ketamine as an add-on treatment, which showed further promise of KT’s safety and effectiveness^47^.

The underlying principle of KT is that the majority of energy is supplied by dietary fat, which is then broken down into ketone bodies for energy production^48^. In the liver, fatty acids are ordinarily converted into acetyl-CoA, which enters the tricarboxylic acid (TCA) cycle. When fatty acid levels are elevated and exceed the metabolic capacity of the TCA cycle, acetyl-CoA is shunted to ketogenesis. Two acetyl-CoAs can combine through a thiolase enzyme to produce acetoacetyl-CoA, a precursor for acetoacetate (ACA) synthesis and β-hydroxybutyrate (BHB). Acetone, the other major ketone body, is produced primarily from spontaneous decarboxylation of ACA and can be eliminated as a volatile substrate through the lungs and kidneys. In the blood, ACA and BHB are transported from the vascular lumen to the brain interstitial space and both glia and neurons by monocarboxylic acid transporters (MCTs). MCT-1 is the principal carrier localized to the vascular endothelium. Within neurons, both ACA and BHB are transported directly into mitochondria, where they are converted to acetyl-CoA via several enzymatic steps. BHB is converted to ACA through D-β-hydroxybutyrate dehydrogenase, and ACA undergoes subsequent conversion to acetoacetyl-CoA through a succinyl-CoA transferase enzyme. Finally, acetoacetyl-CoA-thiolase converts acetoacetyl-CoA to two acetyl-CoA moieties, which then enter the TCA cycle^48^.

Fatty acids can also be transported directly into mitochondria for breakdown and energy production, a process facilitated by the amino acid lysine and methionine-derived carnitine, which can be measured in venous blood samples^49^. The metabolic shift with KT is associated with a variety of central nervous system and general effects on the body. Aside from the ketone bodies enhancing cell energy metabolism by replenishing metabolic reserves, KT has been associated with reducing oxidative stress and inflammatory processes, and regulating key neurotransmitter systems^50–52^, which are all processes implicated in the pathophysiology of AN^15,16,53,54^. Specifically, replacing glucose with ketone bodies via KT to supply the brain with energy enhances γ-aminobutyric acid (GABA) in the brain via enhanced glutamate production converted to glutamine and GABA^55^. GABA is a primary inhibitory neurotransmitter that reduces anxiety^56,57^. In animal models, enhancing systemic ketone levels reduced stress and anxiety-related behaviors^58,59^. GABA function has been reported to be altered in an animal model for AN, and enhancing GABA via ketosis might effectively reduce AN-specific and non-specific anxiety^60,61^. Other studies have found elevated inflammatory markers in AN, and elevating blood ketone levels has been shown to reduce inflammation, which could play a role in the recovery from AN^62^.

Based on those studies, we recently developed a theoretical model that integrates those mechanisms and how KT could be effective for the treatment of AN^63^. We proposed that self-starvation and the resulting state of ketosis may provide an alternative and potentially more efficient energy source for the brain in AN, albeit leading to emaciation and death. However, KT—achieved by a nutrition that primarily relies on fat-derived energy and mimics the metabolic state of starvation without caloric deprivation—could offer therapeutic benefits^63^. The small case series in weight recovered AN demonstrated stable weight and notable improvements across a range of AN-related symptoms, including reductions in eating-related anxiety, weight concerns, and functional impairment^47^. That study was well-tolerated and supported further research. In this trial, we aimed to evaluate the feasibility (ability to recruit participants and conduct study procedures), safety (rate and type of adverse events), acceptability (dropout rate), and effectiveness (symptom change) of KT in a larger sample of individuals with AN who were mildly underweight or had recovered their weight. Specifically, we hypothesized that study participants would be able to complete the treatment and that the intervention would not lead to relapse of AN, such as weight loss, but would lead to improvement in eating disorder symptoms, consistent with findings from the prior case series. We also aimed to examine whether symptom improvement was associated with biological markers of nutritional ketosis, such as blood BHB. In addition, using a naturalistic observational design, we sought to collect pilot data on participants’ continuation of the intervention and to evaluate symptoms and behaviors in relation to their nutritional patterns.

The results of this single-arm study suggest that KT is feasible and well-tolerated in adults with AN who are mildly underweight or weight-restored. The therapy is associated with symptom improvement in both eating disorder-specific and non-specific psychopathology and comorbid conditions, such as depression and anxiety.

## Methods

### Study participants

The UC San Diego Office of IRB Administration approved the study (Trial Registration: NCT06000774, clinicaltrials.gov; registration date August 14, 2023). The primary objectives were: (1) to evaluate the safety and tolerability of ketogenic therapy (KT) in individuals with anorexia nervosa (AN); (2) to assess changes in AN symptoms and behaviors over a 12-week course of KT; and (3) to explore the underlying genetic factors associated with response to KT. The CONSORT flow diagram outlines recruitment and procedures for this single-arm study (Supplementary Fig. 1). During the screening visit, the study investigator met individually with each participant to review the consent form, address any questions, and evaluate the participant’s understanding of the study procedures (Supplementary Fig. 2). Study participants provided informed consent. We recruited individuals aged 18 to 45 years with a history of AN (any subtype) according to DSM-5 criteria, who were, based on population norms, weight-restored or mildly underweight at the time of study initiation (body mass index [BMI] > 17.5 kg/m^2^). This approach allowed minor weight fluctuations in individuals with AN at the lower end of the normal weight spectrum, including those related to fluid shifts. It also effectively included mildly underweight individuals based on BMI. We did not have a target weight for study participants. Determining the correct target BMI for weight restoration in AN can be challenging, and many individuals with AN cannot maintain the recommended weight^64,65^. Therefore, we aimed for weight maintenance.

Power analysis for AN symptoms (EDE-Q Global) based on the previous case series (mean 3.14 ± 1.36 baseline, 1.58 ± 0.88 at 3-months KT) indicated that a sample size of *n* = 20 would provide power > 0.9 at *p* = 0.001^47^. Study participants were recruited from the UCSD Eating Disorder Center after discharge or the community via online advertising. To ensure a significant illness burden, study participants were required to have an elevated Eating Disorder Examination Questionnaire (EDE-Q) Global score > 2.09 for an excellent discrimination validity for AN from healthy control individuals^66–68^. Comorbid unipolar depression, obsessive compulsive disorder, posttraumatic stress disorder and anxiety disorders were acceptable. Antidepressant, anxiolytic, atypical antipsychotic, and mood stabilizers were allowed as they are commonly used and have no specific indication for AN. English had to be the primary spoken language. Exclusion criteria were pregnancy or lactation, electrolyte, blood count, kidney function or liver function abnormalities, psychosis, neurocognitive disorders including dementias or traumatic brain injury that is symptomatic, current alcohol use disorder (AUD) or substance use disorder (SUD) according to DSM-5 criteria, uncontrolled hypertension, hepatic impairment (Class-Pugh b or c), diabetes mellitus, family history of porphyria, history of recent heart attack, vascular disease, or any other current acute medical conditions as determined by the principal investigator, inability or unwillingness to adhere to the TKD diet for the duration of the study, blindness or illiteracy. Subjects had no major medical illness based on medical history and screening labs.

### Study assessments

Subjects were administered the Structured Clinical Interview for DSM-5 (SCID-5, GKWF)^69^. They completed the following self-assessments at the beginning and end of the study: Eating Disorder Examination Questionnaire (EDE-Q)^70^, Eating Disorder Inventory-3 (EDI-3)^71,72^, Eating Disorder Clinical Impairment Assessment (CIA)^73^, Beck Depression Inventory 2 (BDI)^74^, Spielberger State-Trait Anxiety Inventory (STAI)^75^, and Temperament and Character Inventory (TCI)^76^. For weekly symptom monitoring, we adapted the EDE-Q scoring for the past week, as previously done^77^, using only four subscales (Restraint, Eating Concern, Shape Concern, Weight Concern) and retaining the 0-6 scale for comparability with the standard EDE-Q. Mealtime distress (subjective units of distress scale, SUDS) and Trust in Hunger and Fullness Cues (visual analog scale) for the past week were also rated weekly by study participants. During the screening visit only, participants provided blood samples for a complete blood count, a comprehensive metabolic panel, thyroid-stimulating hormone, and a serum pregnancy test to assess their general medical condition.

Exploratory whole-exome DNA sequencing (Supplemental Material, Genetic Analysis) tested for genes involved in energy metabolism that could affect the outcome of KT. Triglycerides and saturated fatty acids are increasingly recognized as important for brain neuronal energy supply, and genetic effects could contribute to inefficient mitochondrial energy creation^78,79^. Carnitine has an important role in transporting fatty acids across the mitochondrial membrane to create energy, and laboratory tests also included blood carnitine levels^80,81^.

### Ketogenic diet induction and maintenance

The study dietitian (SK) introduced participants to the KT, emphasizing three meals and two snacks per day, with macronutrient targets of 70% fat, 20% protein, and 10% carbohydrates, and promoting hunger- and satiety-based eating. Study participants were informed that there was no specific weight goal but that they could not lose weight below a BMI of 17.5. Meals were provided through a ketogenic meal delivery service (Five One Eight Kitchen or a similar approved service), though participants could also prepare their own meals under dietitian guidance. The study covered the food costs. Participants submitted post-meal photographs for the dietitian’s review to estimate consumption as needed. Nutritional ketosis was monitored via daily blood BHB measurements during the initial 14-day induction period, then weekly thereafter (Keto-Mojo)^82^ with a target range of 0.5–3.0 mmol/L, comparable to previous studies^83^. Participants with BHB levels <0.5 mmol/L for two consecutive weeks were to be withdrawn. Body weight was assessed weekly using a home scale (MyClearStep) that enabled blinded data transmission to the study team.

Weekly self-assessments—to monitor AN symptoms, mood, anxiety and suicidality, ketone levels, and weight—were conducted in the evening of the same weekday, prior to meeting with the psychiatrist and dietitian. The qualitative data collected during weekly meetings were not systematically analyzed. Participants received up to three hours of individual peer counseling support (CB). The peer counseling sessions focused on three domains: (1) Ms. Beckwith’s account of her recovery using ketogenic nutrition, (2) guidance on applying KT to daily life and managing challenges, and (3) responses to participant concerns, including fears of weight gain and adverse effects. The UKU Side Effect Rating Scale was administered every 4 weeks to assess adverse effects from the treatment^84,85^.

Three months after the KT intervention, study participants completed questionnaires (EDE-Q, BDI), provided their weight, and met once more with the PI and the study dietitian to review whether they continued KT and to provide qualitative feedback on the study.

### Statistics and reproducibility

Descriptive statistics and two-sided independent t-tests were used to evaluate demographic variables between completer and non-completer individuals. Chi^2^ tests evaluated the frequency of comorbid conditions and medication use. A repeated measures MANCOVA (intent-to-treat analysis, carrying forward data from individuals who dropped out of the study) tested weekly changes over time, including comorbidity (major depressive disorder, obsessive compulsive disorder, posttraumatic stress disorder and generalized anxiety disorder) and medication use (antidepressant and antipsychotic use) in the model. Pearson’s correlations analyzed direct relationships between demographic, symptom change, and blood BHB data. Significant correlations were corrected for multiple comparisons using the false discovery rate^86^. An additional repeated-measures MANCOVA assessed EDE-Q (past month) and BDI scores at pre-, post-, and 3 months post-KT intervention. Post-hoc two-sided independent t-tests or one-way ANOVA compared state and trait variables in completers and non-completers, as well as indicators of successful (EDE-Q and BDI values within normal range) versus non-successful outcomes in the completer group, and a multiple regression analysis tested behavioral variables that predicted AN symptom outcome. Effect sizes are indicated by Cohen’s D (d) or partial eta-squared (ηp²).

#### Inclusion and ethics statement

The research was conducted with local collaborators who fulfilled all authorship criteria as per general ethics standards.

## Results

### Baseline demographic and assessment data

**Fig. 1:**
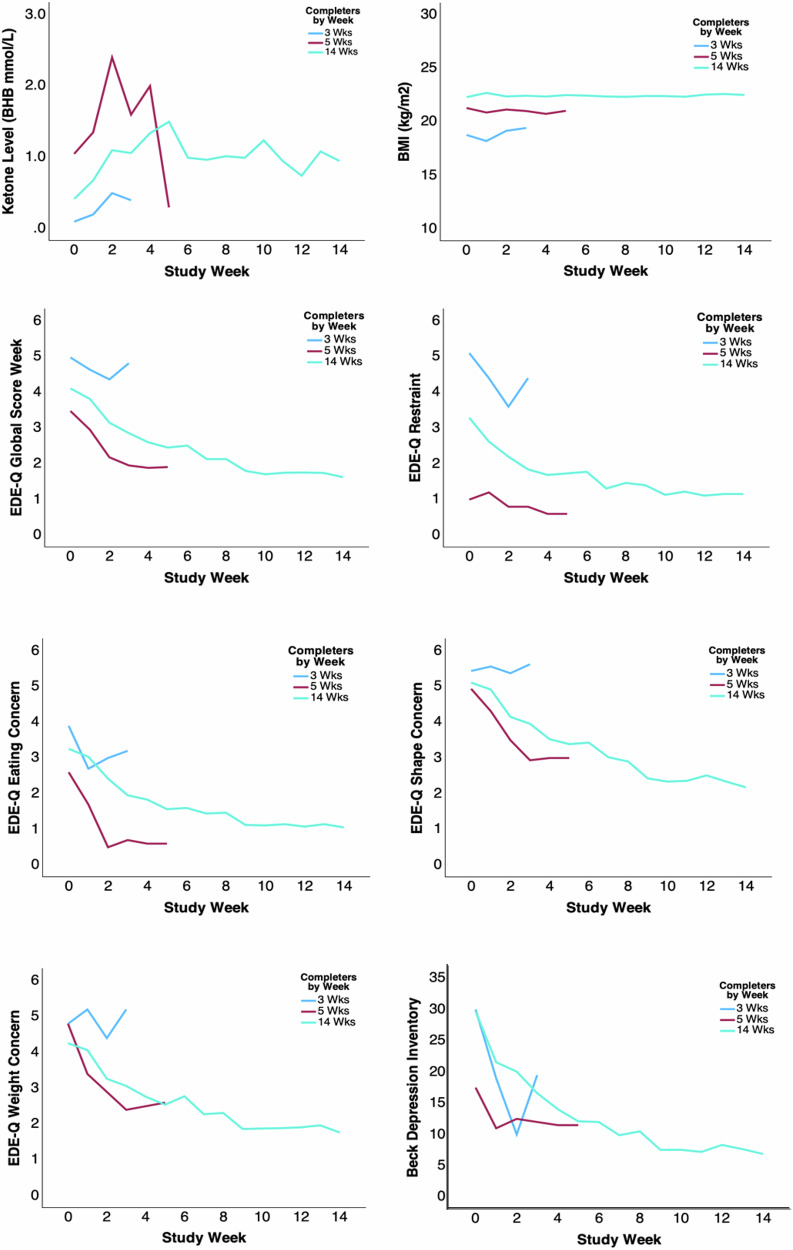
Outcome data by completer and non-completer subgroups. The blue line indicates individuals who dropped out after 2 weeks (*n* = 2), the red line indicates individuals who dropped out after 5 weeks (*n* = 2), and the green line indicates the study completers (*n* = 18). BMI body mass index, EDE-Q Eating Disorder Examination Questionnaire, BDI Beck Depression Inventory.

Study recruitment was conducted from October 2023 to December 2024. Twenty-two individuals (100% female sex; 9% Asian, 9% Asian/White, 82% White; 91% non-Hispanic/Latin) started the KT, 18 of whom completed all 14 weeks (82%); two individuals dropped out after 3, and two after 5 weeks in the study. Non-completers were on average younger, had a shorter duration of illness, and had lower interpersonal alienation scores (Table 1; Fig. 1). Free and total carnitine were lower in completers.

**Table 1:** Group statistics (two-sided t-test) for demographic and behavioral variables for the entire study sample and for completer and non-completer groups

	Full sample (*n* = 22)			Completers (*n* = 18)			Non-completers (*n* = 4)			Completers vs. Non-completers		
	Mean		SD	Mean		SD	Mean		SD	t	*p*	d
Age	24.19	±	5.35	24.98	±	5.59	20.66	±	1.73	−2.746	0.014	−0.832
BMI (kg/m^2^)	21.89	±	3.40	22.30	±	3.54	20.03	±	2.10	−1.220	0.237	−0.674
BMI, Lifetime Low (kg/m^2^)	16.19	±	1.39	16.16	±	1.53	16.31	±	0.33	0.192	0.850	0.106
BMI, Lifetime High (kg/m^2^)	24.20	±	4.00	24.60	±	4.31	22.38	±	1.25	−1.004	0.327	−0.555
Binge Frequency (Weekly)	0.28	±	0.67	0.21	±	0.42	0.63	±	1.25	0.658	0.555	0.671
Purge Frequency (Weekly)	0.00	±	0.00	0.00	±	0.00	0.00	±	0.00	–	–	–
Education (Years)	14.05	±	2.17	4.72	±	1.45	4.50	±	1.00	−0.289	0.775	−0.160
Age of AN Onset (Years)	13.50	±	2.39	13.56	±	2.57	13.25	±	1.50	−0.226	0.823	−0.125
Illness Duration (Years)	10.54	±	5.83	11.26	±	6.19	7.27	±	1.86	−2.304	0.034	−0.693
Global Score Past Month (EDE-Q)	4.13	±	0.79	4.11	±	0.76	4.23	±	1.04	0.260	0.797	0.144
Restraint Past Month (EDE-Q)	3.25	±	1.45	3.29	±	1.24	3.05	±	2.43	−0.191	0.859	−0.162
Eating Concern Past Month (EDE-Q)	3.25	±	1.27	3.26	±	1.24	3.25	±	1.64	−0.008	0.994	−0.004
Shape Concern Past Month (EDE-Q)	5.13	±	0.67	5.11	±	0.65	5.19	±	0.83	0.202	0.842	0.112
Weight Concern Past Month (EDE-Q)	4.35	±	0.87	4.26	±	0.87	4.80	±	0.82	1.142	0.267	0.632
Drive for Thinness (EDI-3)	21.00	±	5.30	20.94	±	5.59	21.25	±	4.43	0.102	0.920	0.056
Bulimia (EDI-3)	6.41	±	6.18	7.00	±	6.44	3.75	±	4.50	−0.950	0.354	−0.525
Body Dissatisfaction (EDI-3)	28.95	±	7.47	29.11	±	7.71	28.25	±	7.27	−0.204	0.841	−0.113
Low Self Esteem (EDI-3)	13.73	±	5.21	14.50	±	5.39	10.25	±	2.36	−1.521	0.144	−0.841
Personal Alienation (EDI-3)	14.18	±	7.02	14.50	±	5.39	8.50	±	4.44	−1.897	0.072	−1.048
Interpersonal Insecurity (EDI-3)	12.68	±	7.50	13.61	±	7.24	8.50	±	8.27	−1.249	0.226	−0.691
Interpersonal Alienation (EDI-3)	10.95	±	5.74	12.33	±	5.34	4.75	±	2.63	−2.731	0.002	−1.510
Interoceptive Deficits (EDI-3)	16.36	±	7.23	16.28	±	7.32	16.75	±	7.93	0.115	0.909	0.064
Emotional Dysregulation (EDI-3)	7.45	±	6.47	8.11	±	6.74	4.50	±	4.66	−1.010	0.325	−0.558
Perfectionism (EDI-3)	17.41	±	4.16	17.33	±	4.24	17.75	±	4.35	0.177	0.861	0.098
Ascetism (EDI-3)	13.50	±	5.45	13.78	±	5.38	12.25	±	6.45	−0.498	0.624	−0.275
Maturity Fears (EDI-3)	15.41	±	8.34	16.67	±	8.18	9.75	±	7.46	−1.550	0.137	−0.857
Clinical Impairment Global (CIA)	31.05	±	10.78	31.72	±	11.59	28.00	±	6.16	−0.615	0.545	−0.340
Personal Impairment (CIA)	14.95	±	3.93	14.83	±	4.29	15.50	±	1.91	0.300	0.767	0.166
Social Impairment (CIA)	8.64	±	4.32	9.11	±	4.28	6.50	±	4.36	−1.100	0.284	−0.608
Cognitive Impairment (CIA)	7.45	±	3.95	7.78	±	4.29	6.00	±	1.15	−0.808	0.429	−0.446
Depression (BDI)	28.59	±	11.48	29.67	±	11.72	23.75	±	10.24	−0.930	0.364	−0.514
State Anxiety (STAI)	49.73	±	8.35	50.67	±	7.31	45.50	±	12.50	−1.126	0.273	−0.623
Trait Anxiety (STAI)	54.27	±	7.89	54.39	±	7.63	53.75	±	10.28	−0.143	0.888	−0.079
Novelty Seeking (TCI)	16.27	±	8.16	16.17	±	7.87	16.75	±	10.72	0.126	0.901	0.070
Harm Avoidance (TCI)	24.55	±	6.45	25.00	±	5.75	22.50	±	9.85	−0.693	0.497	−0.383
Carnitine Free (µmol/L)	30.14	±	7.57	28.06	±	5.99	39.50	±	7.42	3.324	0.003	1.837
Carnitine Total (µmol/L)	39.41	±	8.55	37.22	±	7.50	49.25	±	5.97	2.986	0.007	1.651
Carnitine Esterified (µmol/L)	9.27	±	4.52	9.17	±	4.78	9.75	±	3.59	0.228	0.822	0.126
Carnitine EF Ratio	0.34	±	0.23	0.36	±	0.24	0.28	±	0.15	−0.629	0.536	−0.348
	***N***		**%**	***N***		**%**	***N***		**%**	**χ2**	***p***	
Antidepressant use	8		36	6		33	2		50	0.393	0.531	
Antipsychotic use	2		9	1		6	1		25	1.497	0.221	
Major Depressive Disorder	7		32	6		33	1		25	0.105	0.746	
Obsessive Compulsive Disorder	7		32	5		28	2		50	0.745	0.388	
Posttraumatic Stress Disorder	6		27	6		33	0		0	1.833	0.176	
Generalized Anxiety Disorder	19		86	15		83	4		100	0.772	0.380	

*BMI* body mass index, *EDE-Q* Eating Disorder Examination Questionnaire, *BDI* Beck Depression Inventory, *TCI* Temperament and Character Inventory, *EDI* Eating Disorder Inventory, *STAI* State and Trait Anxiety Inventory, *CIA* Eating Disorder Clinical Impairment Assessment, *EF* esterified-free, *d* Cohen’s D.

Fifty percent of completers and 25% of non-completers (χ2 = 0.825, *p* = 0.364) were in outpatient psychotherapy, 22% of completers and 25% of non-completers were seen by a psychiatrist (χ2 = 0.014, *p* = 0.905), and 56% of completers and 50% of non-completers were previously in a higher level of care (χ2 = 0.041, *p* = 0.840). Half of the study participants started the study by using the meal delivery service, and the other half prepared all meals themselves. Three out of the four non-completers used the meal delivery service only, while the fourth person initially used the service before self-preparing their own meals. Of the 18 completers, no individual used the meal service alone; 10 initially used the meal service before preparing their own meals, while 8 prepared their own meals only. An analysis of meal delivery service use frequency across completer and non-completer groups was not significant.

Of the study non-completers, one decided not to talk to the peer counselor; one person had 1 h, and two had 2 h with the peer counselor. Of the study completers, seven did not meet, ten had 1 h and one had 2 h with the peer counselor. There were no significant group differences in peer counseling hours, and peer counseling hours did not significantly correlate with any outcome variable scores.

Two participants discontinued the intervention at week three. Both presented with relatively high AN symptom severity (Fig. 1), were unable to achieve ketosis, and reported either difficulty meeting caloric requirements or ambivalence about relinquishing the eating disorder. Two additional participants withdrew at week five despite demonstrating a robust ketosis response and comparatively low behavioral symptoms. One participant described the KT as overly restrictive and cited challenges with social eating, whereas the other reported benefit from KT and elected to continue KT independently, without weekly monitoring.

### BMI and behavior outcome variable analysis

**Fig. 2:**
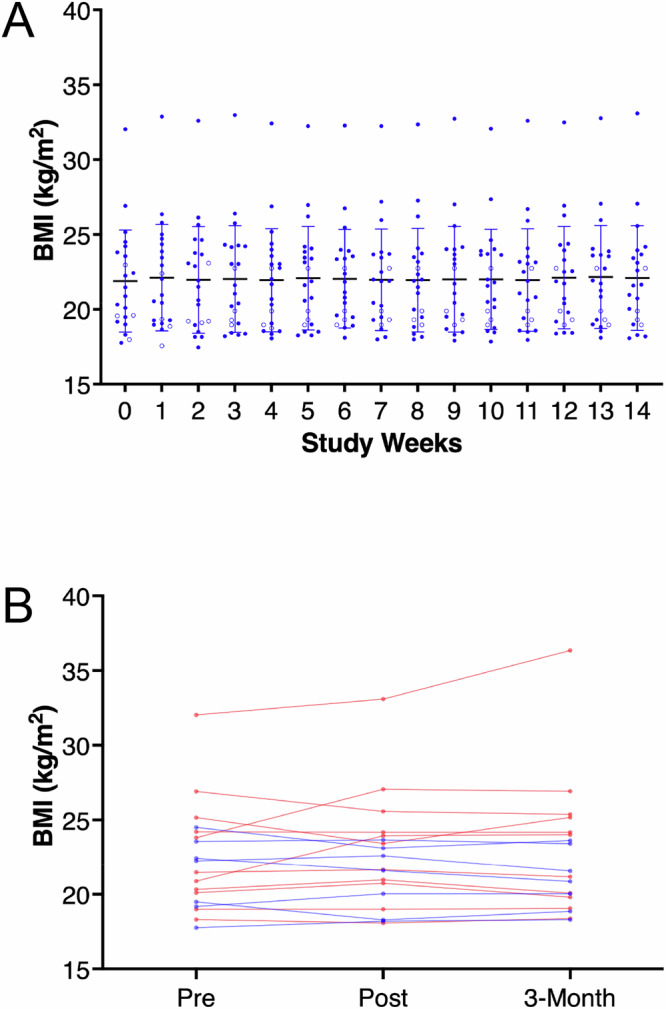
Body mass index (BMI) over time. **A** Individual participant data (*n* = 22) by week, error bars indicate standard deviation centered around the mean; empty circles indicate participants who dropped out (*n* = 4), and data were carried forward in the repeated measures MANCOVA (F = 0.409, *p* = 0.969, ηp² = 0.055, comorbidity and medication use included in the model). **B** Completer data (*n* = 18) pre, post, and 3 months after the KT intervention; blue lines indicate continued KT up to the 3-month assessment.

Change of BMI over the 14 weeks of KT (Fig. 2A) was non-significant in the overall test (Wilk’s lambda = 0.474, *p* = 0.960, ηp² = 0.526) or the within-subject-effects analysis (F = 0.409, *p* = 0.969, ηp² = 0.055). Side effects were initially reported by 40% of individuals and decreased to 0 by study end (Supplementary Fig. 3). No significant adverse events were reported. Adverse effects that were reported were generally mild, including nausea, constipation, or dehydration (Supplementary Fig. 3).

**Fig. 3:**
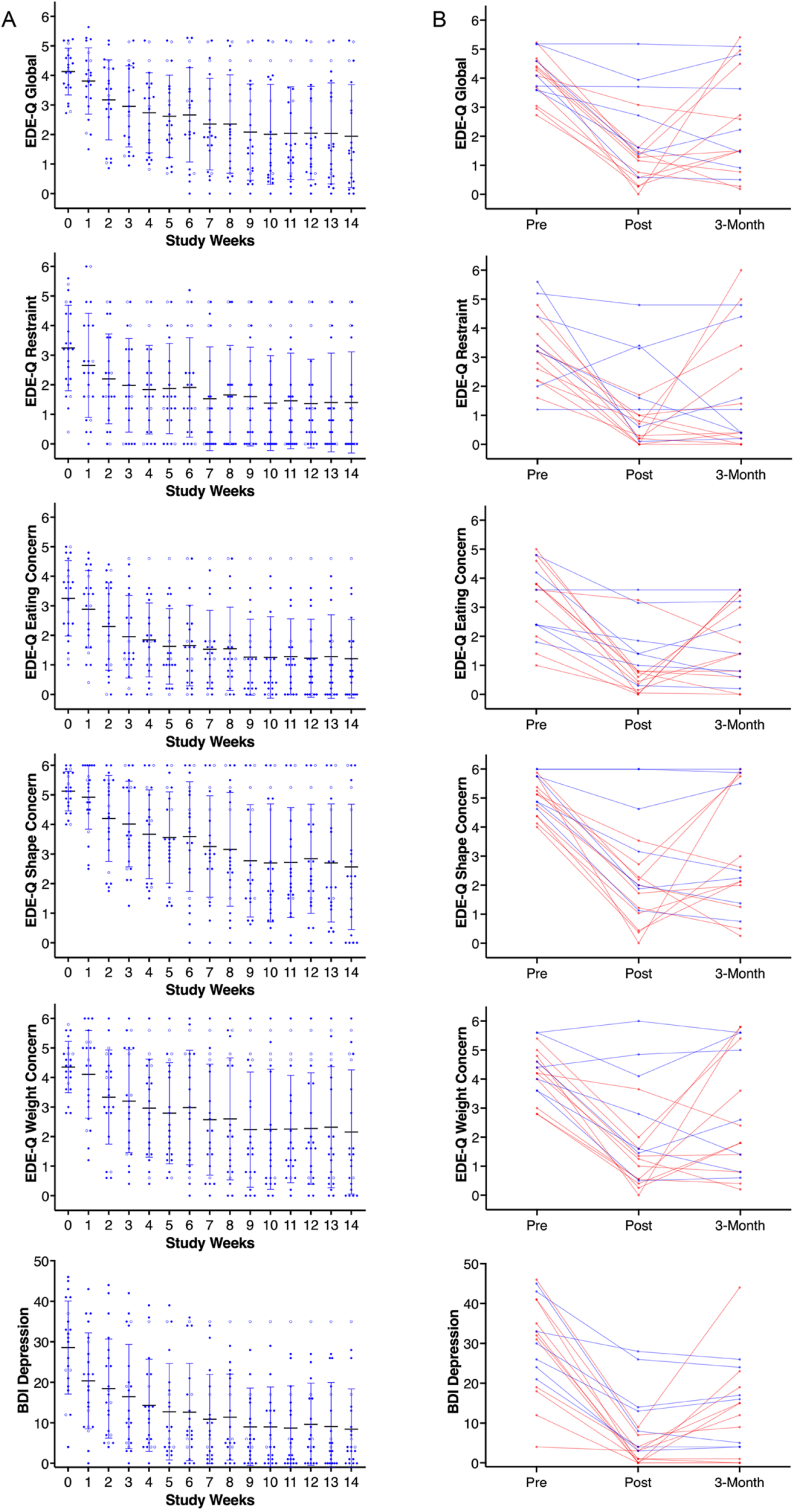
Eating disorder and depression symptom scores over time. **A** Individual participant data (*n* = 22) by week, error bars indicate standard deviation centered around the mean; empty circles indicate participants who dropped out (*n* = 4) and data were carried forward in the repeated measures MANCOVA; EDE-Q Global (F = 12.892, *p* < 0.001, ηp² = 0.674), Restraint (F = 5.081, *p* < 0.001, ηp² = 0.418), Eating Concern (F = 11.316, *p* < 0.001, ηp² = 0.642), Shape Concern (F = 11.443, *p* < 0.001, ηp² = 648) and Weight Concern (F = 8.425, *p* < 0.001, ηp² = 0.588), as well as BDI (F = 10.216, *p* < 0.001, ηp² = 0.599). **B** Subject level completer data (*n* = 18) pre, post, and 3 months after the KT intervention; EDE-Q assessed for the past month; blue lines indicate continued KT up to the 3-month assessment.

The repeated measures MANCOVA for weekly EDE-Q and BDI change over the 14 study weeks (Wilk’s lambda = 0.165, F = 2.383, *p* < 0.001, ηp² = 0.296), showed significant time effects for EDE-Q Global (F = 12.892, *p* < 0.001, ηp² = 0.674), Restraint (F = 5.081, *p* < 0.001, ηp² = 0.418), Eating Concern (F = 11.316, *p* < 0.001, ηp² = 0.642), Shape Concern (F = 11.443, *p* < 0.001, ηp² = 648) and Weight Concern (F = 8.425, *p* < 0.001, ηp² = 0.588), as well as BDI (F = 10.216, *p* < 0.001, ηp² = 0.599) (Fig. 3A). Comorbid conditions and medication did not have significant effects.

An additional repeated measures MANCOVA that analyzed EDE-Q past month for pre-KT, post-KT and at the 3-month follow-up assessment (Fig. 3B), was significant for overall time effects (Wilk’s lambda = 0.085, *p* = 0.042, ηp² = 0.708) showing significant decrease in symptoms for EDE-Q Global (F = 11.439, *p* = 0.003, ηp² = 0.696) at both post-KT (*p* < 0.001) and at the 3-month follow-up assessment (*p* < 0.001) versus pre-KT; EDE-Q Restraint (F = 6.212, *p* = 0.018, ηp² = 0.554), with lower post-KT (*p* < 0.01) and 3-month (*p* < 0.001) versus pre-KT; EDE-Q Eating Concern was lower (F = −7.637, *p* = 0.010, ηp² = 0.604) for post-KT (*p* < 0.003) and at 3-months (*p* < 0.007) versus pre-KT; EDE-Q Shape Concern was lower (F = 15.030, *p* = <0.001, ηp² = 0.750) for post-KT (*p* < 0.001) and at 3-months (*p* < 0.002) versus pre-KT; EDE-Q Weight Concern was lower (F = 7.523, *p* = 029, ηp² = 0.601) for post-KT (*p* < 0.001) and 3-months (*p* < 0.007) versus pre-KT. Similarly, for BDI, there was a significant time effect (Wilk’s lambda = 0.085, *p* = 0.007, ηp² = 0.915), showing a significant decrease in symptoms (F = 15.814, *p* < 0.001, ηp² = 0.760) at both post-KT (*p* < 0.001) and 3-month (*p* < 0.002) versus pre-KT. Body mass index (BMI) from pre-KT to post-KT or 3-month follow-up did not significantly change as indicated by the overall statistics (Wilk’s lambda = 0.846, *p* = 0.715, ηp² = 0.154) and within-subject effects results (F = 0.050, *p* = 0.951, ηp² = 0.010; Fig. 2B). Inclusion of continued KT use beyond the 14-week study period as a predictor was not significantly associated with any outcome variables.

The pre-post KT paired-samples t-tests in completers indicated significant improvements in EDI-3, CIA, STAI, and TCI Harm Avoidance (Table 2). An analysis comparing outcome variables at the end of the study between those who prepared their own meals throughout the study versus those who initially used the meal delivery service before preparing their own meals was non-significant for EDE-Q Global (F = 0.003, *p* = 0.954, ηp² = 0.000) and BDI Depression (F = 0.607, *p* = 447, ηp² = 0.037) scores).

**Table 2:** Change of behavioral variables from baseline to after ketogenic therapy (KT) using a two-sided paired t-test in study completers

	Completers (*n* = 18)									
	Pre KT			Post KT						
	Mean		SD	Mean		SD		t	*p*	d
BMI (kg/m^2^)	22.30	±	3.54	22.51	±	3.69		0.660	0.518	-0.155
Binge Frequency (Weekly)	0.21	±	0.42	0.00	±	0.00		2.093	0.052	0.493
Drive for Thinness (EDI-3)	20.94	±	5.59	13.56	±	8.60		4.829	0.0002	1.138
Bulimia (EDI−3)	7.00	±	6.44	2.28	±	3.08		3.772	0.002	0.889
Body Dissatisfaction (EDI−3)	29.11	±	7.71	18.83	±	11.26		5.442	0.00004	1.283
Low Self Esteem (EDI-3)	14.50	±	5.39	7.33	±	6.96		5.549	0.00004	1.308
Personal Alienation (EDI-3)	15.44	±	6.94	8.44	±	5.96		4.999	0.0001	1.178
Interpersonal Insecurity (EDI-3)	13.61	±	7.24	9.28	±	5.99		2.721	0.015	0.641
Interpersonal Alienation (EDI-3)	12.33	±	5.34	8.28	±	4.52		4.294	0.0005	1.012
Interoceptive Deficits (EDI-3)	16.28	±	7.31	10.22	±	6.97		3.428	0.003	0.808
Emotional Dysregulation (EDI-3)	8.11	±	6.74	5.00	±	5.28		3.486	0.003	0.822
Perfectionism (EDI-3)	17.33	±	4.24	14.44	±	5.29		3.790	0.001	0.893
Ascetism (EDI-3)	13.78	±	5.37	8.83	±	5.39		5.142	0.00008	1.212
Maturity Fears (EDI-3)	16.67	±	8.17	10.56	±	8.35		6.557	0.000005	1.546
Clinical Impairment Assessment Global (CIA)	31.72	±	11.59	14.83	±	12.63		6.234	0.000009	1.469
Personal Impairment (CIA)	14.83	±	4.29	7.28	±	6.10		6.269	0.000008	1.478
Social Impairment (CIA)	9.11	±	4.28	3.89	±	3.95		5.429	0.00005	1.280
Cognitive Impairment (CIA)	7.78	±	4.29	3.67	±	3.33		4.703	0.0002	1.109
Depression (BDI)	29.67	±	11.72	6.89	±	8.49		8.135	0.0000003	1.917
State Anxiety (STAI)	50.67	±	7.31	39.11	±	9.24		4.468	0.0003	1.053
Trait Anxiety (STAI)	54.39	±	7.63	40.50	±	11.79		4.625	0.0002	1.090
Novelty Seeking (TCI)	16.17	±	7.87	17.50	±	8.28		1.551	0.139	-0.366
Harm Avoidance (TCI)	25.00	±	5.75	20.83	±	6.32		2.926	0.009	0.690

Ketone level changes over time (F = 1.482, *p* = 0.132, ηp² = 0.181) were non-significant (Supplementary Fig. 4). Trust in Hunger and Fullness Cues significantly increased (F = 6.763, *p* < 0.001, ηp² = 0.470), and Mealtime Distress SUDS (F = 5.063, *p* < 0.001, ηp² = 0.399) significantly decreased. Percent Meal Completion (F = 0.974, *p* = 0.479) was non-significant over time.

### Successful versus non-successful completers

**Fig. 4:**
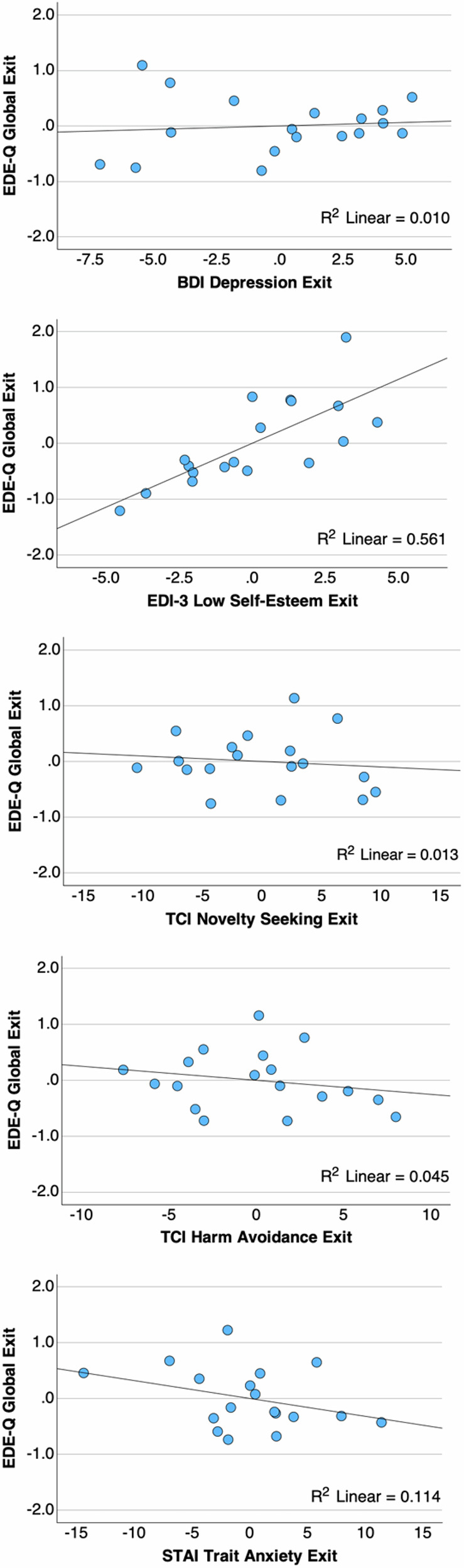
Multiple linear regression analysis, partial correlation plots, testing the effects of depression, low self-esteem, Novelty seeking, harm avoidance, and trait anxiety on Eating Disorder Examination Questionnaire Global score at exit. Among the independent variables, EDI-3 Low Self-esteem was significant (beta = 0.229, standardized beta = −1.108, *p* = 0.002), but the other variables tested were not.

Seventy-two percent (*n* = 13) of the completers (59% of the full sample) had an EDE-Q Global score in the recovered range at study end (Supplementary Tables 1 and 2). A one-way ANOVA explored whether state or trait behaviors (TCI, EDI-3, BDI, and STAI) could differentiate groups. Participants whose EDE-Q Global scores did not normalize and fall within normal range below 2.09 at the end of the study had, at baseline, lower Novelty Seeking (TCI) and elevated Low Self-Esteem (EDI-3) scores. At the end of the study, this group continued to display lower Novelty Seeking and elevated Harm Avoidance (TCI), Depression (BDI), and Low Self-Esteem (EDI-3) ratings (Supplementary Table 2). A multiple regression analysis using the end-of-study group data, separating EDI-3 Low Self-esteem, TCI Novelty Seeking and Harm Avoidance, BDI Depression, and STAI Trait Anxiety as independent variables, and exit EDE-Q Global as the dependent variable, showed a significant overall effect (F = 16.713, *p* < 0.001). Between independent variables, EDI-3 Low Self-esteem was significant (beta = 0.229, standardized beta = −1.108, *p* = 0.002), while Novelty Seeking (beta = −0.010, standardized beta = −0.056, *p* = 0.697), Harm Avoidance (beta = 0.025, standardized beta = 0.110, *p* = 0.469), Depression (beta = 0.013, standardized beta = 0.075, *p* = 0.740) and Trait Anxiety (beta = −0.032, standardized beta = −0.262, *p* = 0.237) were not (Fig. 4; Supplementary Fig. 5).

### Correlation analyses

Baseline total carnitine correlated positively with baseline ketone levels (r = 0.436, *p* = 0.043). Weekly ketone levels after establishing ketosis were positively associated with weekly EDE-Q and BDI scores, but negatively with weekly scores for subjective trust in Hunger and Satiety Cues (Table 3; Supplementary Figs. 6, 7 and 8).

**Table 3:** Correlations (Pearson’s correlation coefficient, r) between weekly ketone levels and behavior assessment data

Correlation Ketone level and behavior by week	EDE-Q global		EDE-Q restraint		EDE-Q eating concern		EDE-Q shape concern		EDE-Q weight concern		Trust in hunger and satiety cues		BDI depression	
	r	*p*	r	*p*	r	*p*	r	*p*	r	*p*	r	*p*	r	*p*
0 (22)	0.033	0.883	0.075	0.741	−0.115	0.609	0.064	0.779	0.180	0.423	−0.193	0.388	0.217	0.332
1 (22)	−0.013	0.953	−0.053	0.814	−0.065	0.773	0.016	0.942	0.102	0.653	0.221	0.322	0.142	0.528
2 (22)	−0.149	0.509	−0.177	0.431	−0.235	0.293	−0.109	0.630	−0.003	0.988	0.101	0.655	−0.068	0.764
3 (22)	0.392	0.071	0.383	0.078	0.219	0.328	0.351	0.109	0.448	0.036	−0.407	0.060	0.401	0.065
4 (20)	0.229	0.332	0.306	0.189	0.048	0.840	0.196	0.407	0.249	0.291	−0.118	0.620	0.331	0.154
5 (20)	**0.571**	**0.009**	**0.581**	**0.007**	0.434	0.056	**0.561**	**0.010**	0.493	0.027	**−0.642**	**0.002**	0.347	0.134
6 (18)	**0.684**	**0.002**	**0.730**	**0.001**	0.54	0.021	**0.645**	**0.004**	0.565	0.014	**−0.655**	**0.003**	**0.604**	**0.008**
7 (18)	**0.574**	**0.013**	0.541	0.020	0.529	0.024	0.475	0.047	0.591	0.010	−0.456	0.057	**0.613**	**0.007**
8 (18)	0.503	0.033	**0.585**	**0.011**	0.479	0.044	0.416	0.086	0.451	0.060	−0.440	0.067	0.435	0.071
9 (18)	**0.561**	**0.015**	0.488	0.040	0.508	0.031	**0.563**	**0.015**	0.535	0.022	**−0.545**	**0.019**	0.388	0.111
10 (18)	0.414	0.087	0.417	0.085	0.362	0.140	0.410	0.091	0.362	0.140	−0.279	0.262	0.185	0.464
11 (18)	**0.578**	**0.012**	0.369	0.132	0.536	0.022	**0.605**	**0.008**	0.574	0.013	**−0.591**	**0.010**	0.285	0.251
12 (18)	**0.578**	**0.012**	0.514	0.029	0.600	0.009	0.509	0.031	0.541	0.020	**−0.650**	**0.003**	0.165	0.514
13 (18)	0.403	0.098	0.247	0.324	0.239	0.340	0.482	0.043	0.428	0.076	−0.408	0.092	0.455	0.058
14 (18)	**0.749**	**0.0004**	**0.630**	**0.005**	**0.747**	**0.0004**	**0.682**	**0.002**	0.762	**0.0002**	**−0.622**	**0.006**	**0.624**	**0.006**

Bolded numbers indicate values that remained significant after false discovery rate multiple comparison correction.

### Three-month follow-up

At the 3-month follow-up, 39% (*n* = 7) continued the ketogenic diet, and 61% (*n* = 11) did not (Fig. 3B). Four of the five (80%) whose EDE-Q scores had not normalized at the end of KT (non-successful completers) continued KT, but only three (20%) of the thirteen with EDE-Q scores in the normal range (successful completers) continued KT. At the 3-month assessment, two of the seven individuals (28%) who continued KT had an increase in EDE-Q symptom scores, but seven (64%) of those who did not continue KT had increasing EDE-Q scores.

### Exploratory genetic analysis

We explored genes that are frequently associated with carnitine deficiency and metabolism and contribute to carnitine transport into cell bodies and mitochondria, SLC22A5, CPT1, and CPT2^87–89^. Exploratory whole-exome DNA sequencing (Supplemental Material) indicated that total carnitine levels in individuals with a CPT2 gene mutation (*n* = 8, 44.5 ± 7.9) versus those without (*n* = 14, total carnitine = 36.5±7.7) were significantly higher (t = −2.321, *p* = 0.031, d = −1.029, CI95% = −1.942 to −0.093), and free carnitine levels were lower in those with the SLC22A5 mutation (*n* = 2, 19.0 ± 5.7) versus those without (*n* = 20, 31.3 ± 6.9) the mutation (t = 2.420, *p* = 0.025, d = 1.795, CI95% = 0.220 to 3.330).

## Discussion

This study in individuals with AN at a BMI above 17.5 suggests that KT is well-tolerated and associated with a fast reduction in both eating disorder-specific and depressive symptoms. Depression scores improved in all, and EDE-Q and EDI-3 eating disorder symptom scores were within the normal range at study end for most completers. Elevated low self-esteem scores distinguished those with poor improvement in AN symptoms. Weekly ketone levels were significantly associated with eating disorder and depression severity scores. The study had a high completion rate, and physiological factors were significantly different between completers and non-completers, including baseline carnitine levels, suggesting altered metabolism.

Weight restoration and maintenance within a healthy range are central to the treatment for AN. The antipsychotic medication olanzapine has shown modest additional benefits, and novel treatments such as ketamine, psilocybin, or neuromodulation are being explored. However, no biological intervention has yet been approved for the treatment of AN^90,91^. Importantly, cognitive and emotional symptoms often persist even after successful weight restoration, contributing to a high risk of relapse^4–6,10,92–94^. This well-powered study supports and extends the results from the previous case series^47^. Study participants as a whole established ketosis within 2 weeks and maintained this state over the study duration. The mean baseline EDE-Q Global score in our sample (4.11) was comparable to other study populations with severe symptoms^95^, and decreased to below the cutoff for AN at study end (1.72). This level of improvement over a relatively short period of time is not typically seen in treatment for AN. For instance, previously reported EDE-Q Global scores for patients with AN across partial hospital programs changed from 4.08 at intake to 3.09 at discharge^96^.

Our subject-level data indicated that 72% of completers were well below the EDE-Q clinical cutoff, while EDE-Q scores remained high in 28% of participants. Conversely, all participants showed improved depression scores at the end of the study. The post-hoc analysis indicated that, among the factors distinguishing successful from unsuccessful completers, low self-esteem was associated with a higher EDE-Q Global score. At the same time, the effects of depression, trait anxiety, harm avoidance, and novelty seeking were not significant in the multiple regression analysis. Low self-esteem has been associated with AN in the past, even in the absence of depression, suggesting trait abnormalities in AN that have profound effects on treatment outcome, and treatments that target low self-esteem in AN should be further developed and implemented in treatment^97–99^.

Recently, we developed a model of how a ketogenic diet could support symptom recovery in AN, but the underlying mechanisms of KT’s effects on AN remain elusive^63^. In our study, total carnitine was positively associated with baseline BHB levels, consistent with some but not all previous studies^100^. While the study sample was small, there were significant effects of carnitine cycle genes on total or free carnitine levels, indicating that those genes are important in achieving the ketosis state. Ketone levels rose over the first 4 weeks, then decreased slightly and stabilized from week six to fourteen. EDE-Q ratings decreased after week one and tended to stabilize after week nine. Depression ratings decreased and stabilized also at week nine. This pattern suggests that the biological effects of KT reached a plateau after approximately 2 months of ketosis in this study sample. Gradual effect changes of the KT have been reported in neurology for seizure control^101,102^. Lack of ketosis and associated high symptoms in week three dropouts and low symptoms together with high ketosis in the week five dropout group, suggest an inverse relationship between ketosis and symptom severity. However, weekly BHB levels were positively correlated with EDE-Q and BDI ratings, indicating higher ketosis and worse symptomatology, while trust in hunger and satiety cues correlated negatively. Those results were unexpected. Some neurological research suggested that higher BHB levels are associated with better seizure control, and initially, we hypothesized an inverse linear relationship between BHB levels and eating disorder or mood symptoms^103^. More recent research indicates that the relationship between BHB, a measure of ketosis, and symptoms is more complex, possibly non-linear, and may take the form of an inverted-U curve^104^. Future studies will indicate whether individuals with more severe symptomatology are more susceptible to changes in ketone levels, or whether, for instance, a low level of ketosis is most beneficial.

Eighty percent of study participants completed the study, suggesting overall good tolerance. Side effects were intermittent and largely inconsistent, except for increased nausea when at a BHB level above three mmol/L. Some individuals reported intermittent constipation, others reported less bloating and constipation on KT. No serious adverse effects were reported (Supplemental Material). Thirty-nine percent of study completers continued KT at the 3-month follow-up, indicating good tolerance of the intervention and motivation to continue KT.

There is concern in the eating disorder field that dietary interventions promoting patterns perceived as restrictive or unbalanced may pose risks to this vulnerable population. In particular, the ketogenic diet is often portrayed as a weight loss regimen, raising fears that such an approach could trigger weight loss or relapse in individuals with anorexia nervosa (AN). However, these concerns were not supported by the present findings. During the intervention, 13 of the 18 completers maintained their weight within 1 BMI point of baseline; 4 participants experienced modest weight loss (1.3–1.7 BMI points); and 3 participants showed weight gain (1.1–3.2 BMI points). On average, participants showed a slight increase in BMI of 0.2, and no participant’s BMI fell below 17.5. Regarding absolute weight change (in kilograms), independent of height, the mean change was an increase of 0.5 kg, with a median increase of 0.4 kg. Six participants remained within ±1 kg of their baseline weight, five lost between 2.7 kg and 4.3 kg, and seven gained between 1.2 kg and 8.7 kg. Individualized meal plans were developed by the study dietitian using visual portion sizes rather than calorie counts, allowing participants to eat according to comfort and hunger cues. However, they were instructed to eat more if they were losing weight. Because meals were consumed at home without direct supervision, participants retained the option to restrict intake if desired. Some initially reported significant anxiety and required extensive coaching to ensure adequate intake; however, trust in the dietary intervention improved over time. Overall, while KT initially provoked anxiety, it effectively supported weight maintenance without evidence of relapse or worsening of symptoms.

The study did not prescribe a target weight; instead, the hypothesis was that by normalizing metabolism and energy utilization, participants would move toward more intuitive eating, allowing weight stabilization around their genetically determined set point. Anecdotal comments from study completers did not support concerns but for instance included such as “I wish I had known about this treatment years ago,” “I did not think I could change,” and “I got my confidence back,” but also acknowledged “in the beginning I did not think I would make it through,” or “eating fat was very hard.” Some study participants also tested the effects of the KT, for instance, when on vacation, not being as strict with the ketogenic nutrition, but noticed the “difference immediately with feeling more lethargic.”

Another potential concern raised was whether KT would decrease the fear of eating due to the low carbohydrate intake, while increasing the fear of eating carbohydrates. Study participants did not report this. Fat is calorically dense and typically more fear-inducing, and past research indicates that individuals with AN eat relatively less fat and more carbohydrates than healthy controls^105^. Reintegration of carbohydrates was commonly associated with feeling more “sluggish,” and some individuals reported increased body aches that had subsided during the KT intervention.

In general, most individuals’ comments reflected surprise that this intervention could provide a level of recovery that they had not experienced in the past. However, two of the study participants could not eat sufficient ketogenic food to establish ketosis, and one person found the protocol too restrictive, especially in social settings, indicating adherence and tolerability challenges for some individuals, and younger age and shorter illness duration may potentially be associated with non-completion.

With respect to limitations, although the study was sufficiently powered to detect significant effects, the sample was small, limited to females, and predominantly White and non-Hispanic, which restricts the generalizability of the findings. Future research should replicate these findings in more diverse populations and incorporate objective assessments of brain function, such as metabolic PET imaging, to assess brain glucose metabolism. The KT intervention was a single-arm study without a placebo control. Individuals with AN have typically undergone many restrictive or non-restrictive meal plans throughout their lives, but none of those have provided symptom relief and often have increased symptoms. Eating fats is particularly difficult and anxiety-provoking for this population, which was reflected in increased initial weight concerns. The study results may have been affected by several factors that were not controlled for, including expectation or placebo effects, changes to medication or outpatient provider contacts, or eating behaviors not captured in the assessments. No extensive pre- or post-intervention labs, including lipid profiles, were included in this study, limiting the conclusions that can be drawn regarding nutritional adequacy. Three individuals were in the low-weight, mild AN category, based on DSM-5 criteria. Two of those were in the study completer group (BMI: 17.8, 18.3) and one person in the non-completer group (BMI: 18.0; χ2 = 0.536, *p* = 0.470). We believe that including those individuals and a broader BMI range strengthens the study’s results, indicating that the intervention may be suitable for at least some individuals in the mild AN category. Six participants experienced brief episodes during which BHB levels dropped below 0.5 mmol/L. These transient departures from ketosis occurred in the context of intercurrent illness, inadvertent consumption of sugar-containing medications or supplements, menstruation, or alcohol intake. An exploratory correlation analysis examining changes in BHB levels in relation to AN or depressive symptoms did not yield significant associations. However, these null findings may have been influenced by the limited sample size. The observed correlations between ketone levels and behavioral data across time points support the validity of the association, but the exact nature of this relationship remains unclear. We modified the EDE-Q for weekly measurements, as previously done^77^. Cronbach’s alpha for the EDE-Q adapted for weekly measures yielded 0.8 for baseline and week one, and 0.9 for weeks 2 to 14, indicating good to excellent consistency. The study sample was small for a genetic analysis, but genotype significantly affected BHB levels and provides direction for future research. Many study participants were connected to an outpatient provider. Participants concurrently receiving outpatient psychotherapy typically reported weekly therapy sessions that were supportive or cognitive-behavioral oriented. However, the specific influence of this therapy on the study outcomes was not systematically assessed. No study participant was in a higher level of care. Study participants intermittently took medium-chain triglyceride oil, and five individuals took a carnitine supplement due to low baseline levels; however, those factors did not have a significant effect on outcome variables. Non-completers used, on average, more hours with the peer counselor than completers, while using the meal delivery service versus preparing meals themselves was not statistically significant; however, these results should be viewed with caution due to the small sample size.

This study demonstrates that the KT is well-tolerated and highly effective in reducing eating disorder and depression symptomatology in women with AN, except for individuals exhibiting markedly low self-esteem. Although objective biomarkers of ketosis were associated with eating disorder and depression severity, the precise nature of these associations and mechanisms remains uncertain. These findings support KT as a promising therapeutic intervention in mildly underweight or weight-normalized AN and warrant testing in a controlled study design, comparing the intervention with, for instance, the Mediterranean diet, which has been tested previously in another population^106^. Further research is warranted to also evaluate efficacy of KT across populations with eating disorders and to elucidate its neurobiological mechanisms of action.

## Supplementary information

Supplemental Material

Description of Additional Supplementary files

## Source data

Source Data

## Data Availability

The Source data are included in the Manuscript’s Supplementary data files. The Study Protocol is available to readers upon reasonable request.
