# Supplementary material for: Symptom impact and safety of ketogenic therapy in adults with anorexia nervosa: a feasibility trial
Source: Commun Med (Lond). 2026 Jun 3;6:315. doi: 10.1038/s43856-026-01644-0 (PMC13234144; doi:10.1038/s43856-026-01644-0)

Supplemental Material

Supplemental Figure 1. CONSORT Flow Diagram of the phases of the study, modified for a non-randomized trial.

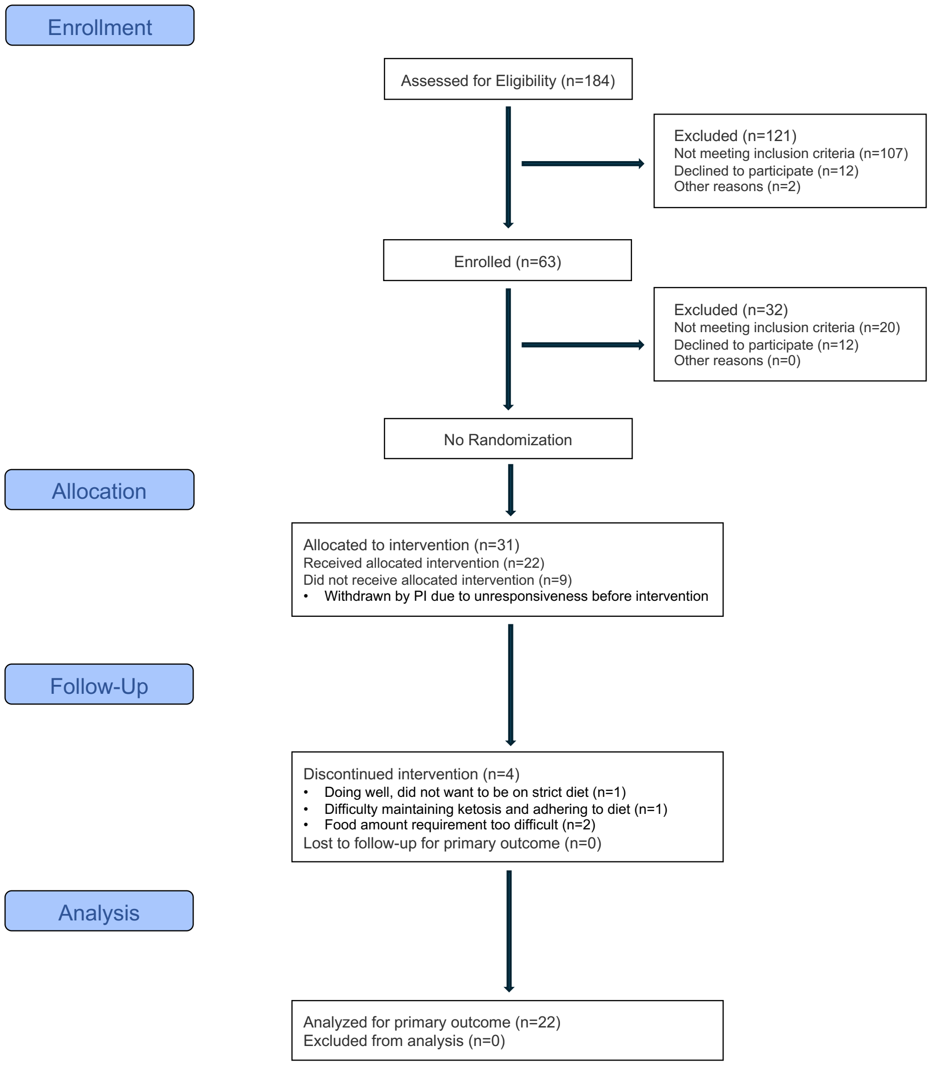

Supplemental Figure 2. Schematic of study procedures.

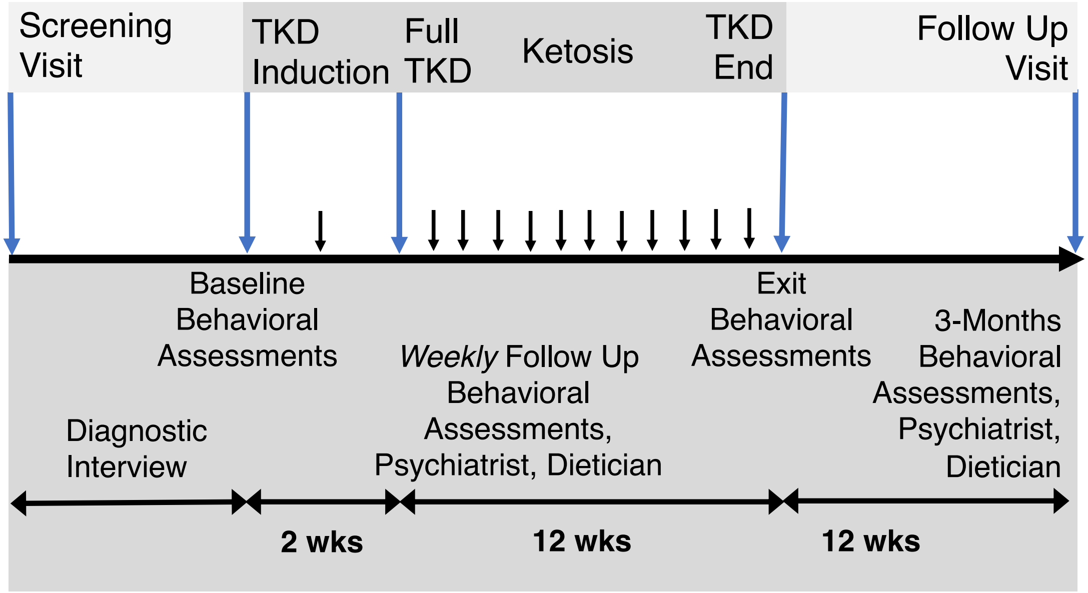

Supplementary Methods

Whole Exome Sequencing: Buccal swabs were collected using iSWAB-Discovery (#ISAWB-DSC, Mawi DNA Technologies LLC, Pleasanton, CA). Samples were sent to Novogene (Sacramento, CA) for clinical whole exome sequencing (100X coverage). Genomic DNA was randomly sheared into short fragments with a size range of 180-280 base pairs. These fragments were end repaired, A-tailed, and further ligated with Illumina adapters. The fragments with adapters were PCR amplified, filtered by size, and purified. Hybridization capture of libraries proceeded as follows: the prepped libraries were hybridized with biotin-labeled probes and precipitated with magnetic beads conjugated to streptavidin to capture the exons. Subsequently, non-hybridized fragments were washed out and probes were digested. The captured libraries were enriched by PCR amplification. The library was checked with Qubit and real-time PCR for quantification and for detection of size distribution. Quantified libraries are pooled and sequenced on Illumina platforms the PE150 protocol, according to effective library concentration and data amount required.

Quality Control and Bioinformatics Analysis Pipeline- Bioinformatic analysis includes: 1) Data quality control by filtering out reads containing adapters or with low quality; 2) Alignment with reference genome, statistics of sequencing depth and coverage;3) SNP/InDel calling, annotation and statistics; and 4) Somatic SNP/InDdel/CNV calling, and annotation. The original fluorescence image files obtained from the sequencing platform are transformed to short reads (Raw data) by base calling. These short reads are recorded in FASTQ (.fq) format files, which contain sequence information (reads) and corresponding base quality. Novogene performed quality control according to following procedures: Discarded paired-end reads if either one read contains adapter contamination (>10 nucleotides aligned to the adapter, allowing ≤ 10% mismatches); Discarded paired-end reads if more than 10% of bases are uncertain (read as N)

in either one read; Discard paired-end reads if the proportion of low-quality (Phred quality < 5) bases is over 50% in either one read.

Burrows-Wheeler Aligner (BWA) was utilized to map the paired-end clean reads to the human reference genome (hg38, http://hgdownload.cse.ucsc.edu/goldenPath/hg38/bigZips/analysisSet/hg38.analysisSet.2bit). The original mapping result is obtained in BAM format, Sambamba was then used for sorting the BAM files and Picard is utilized to mark duplicate reads. Final BAM files are obtained after these steps and the coverage and depth is computed based on BAM. Germline mutations are detected for single nucleotide polymorphisms (SNPs) and small insertions and deletions that are less than 50 bp in length (InDels). Following genomic variant detection, annotation of variants with the tool ANNOVAR.

Supplemental Figure 3. Side effect frequencies over time

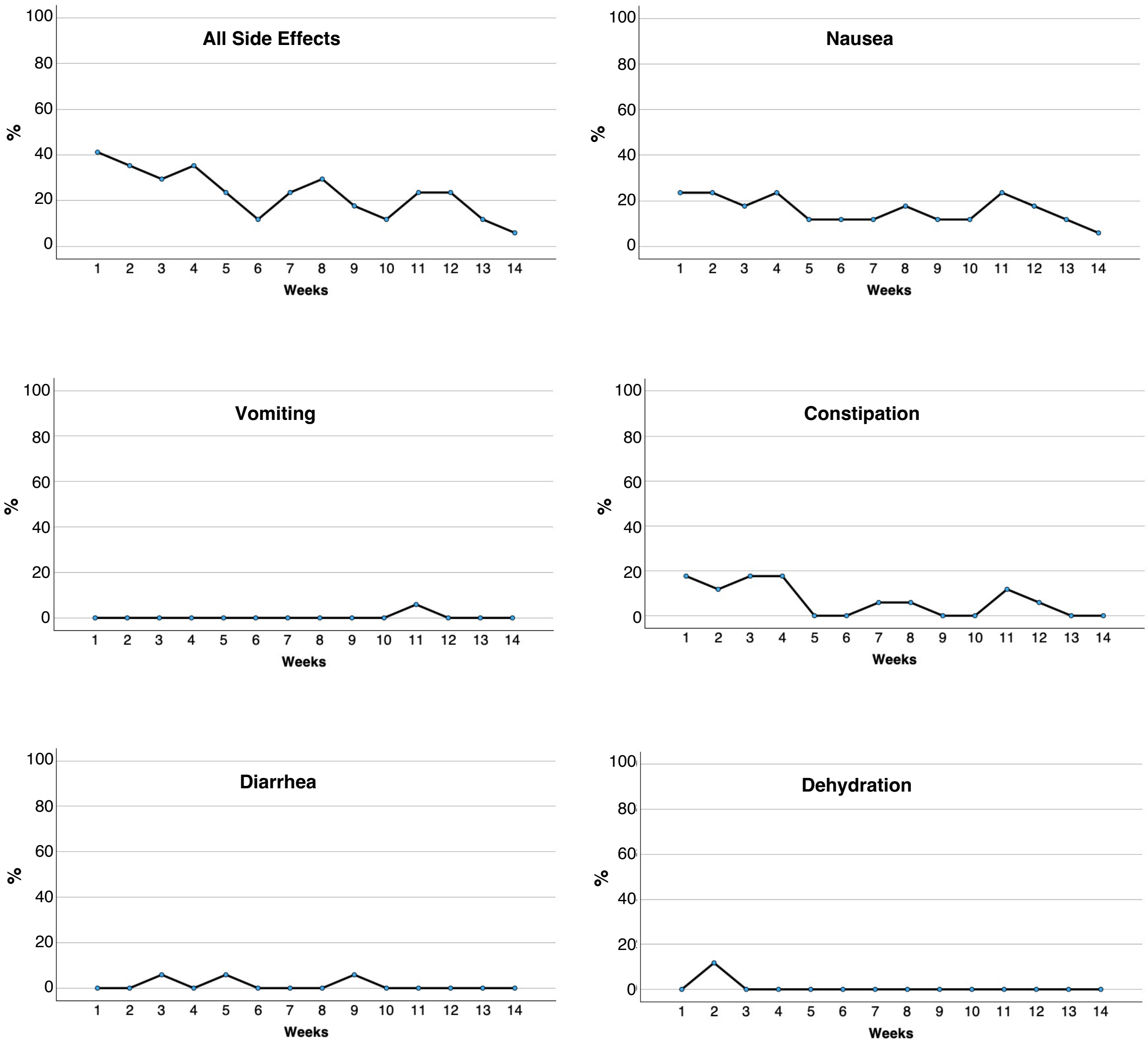

Supplemental Figure 4. Ketone levels, percent meal completion, mealtime distress and trust in hunger and satiety cues over time.

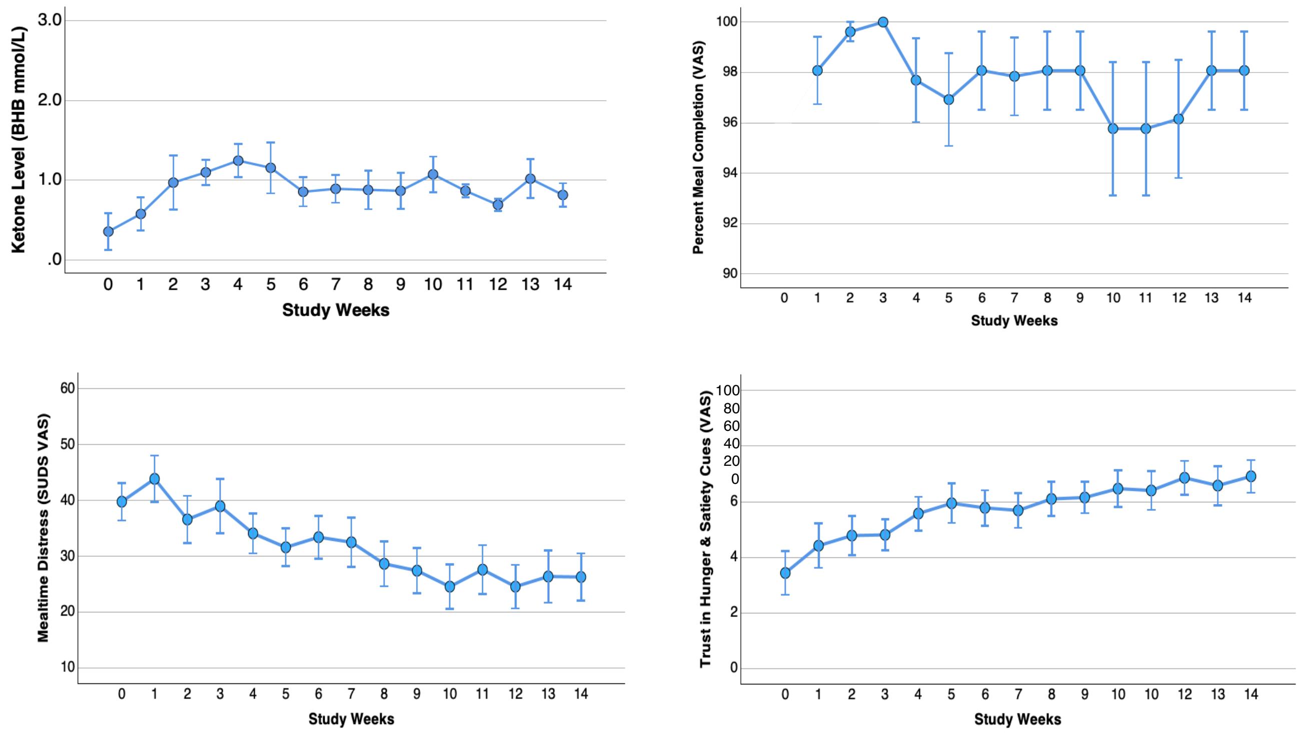

Supplemental Table 1. Comparison of successful versus non-successful completers on non-eating disorder trait variables (two-sided t-test)

	EDE-Q Global < 2 (n=13)		EDE-Q Global > 2 (n=5)		t	p	d	95% Confidence Interval	
	M	SD	M	SD				Lower	Upper
NS sum baseline	18.69	7.35	9.60	5.13	-2.517	**0.023**	**-1.325**	**-2.435**	**-0.180**
HA sum baseline	24.00	6.28	27.60	3.21	1.206	0.246	0.634	-0.429	1.679
RD sum baseline	13.31	4.37	15.20	5.07	0.790	0.441	0.416	-0.632	1.451
P sum baseline	5.23	1.64	5.20	2.05	-0.033	0.974	-0.018	-1.049	1.014
NS sum exit	20.15	7.19	10.60	7.37	-2.510	**0.023**	**-1.321**	**-2.431**	**-0.177**
HA sum exit	18.85	5.98	26.00	4.00	2.447	**0.026**	**1.288**	**0.149**	**2.393**
RD sum exit	15.23	4.13	15.20	6.76	-0.012	0.991	-0.006	-1.038	1.025
P sum exit	5.08	1.55	5.80	1.30	0.920	0.371	0.484	-0.568	1.521
BDI baseline	28.46	13.26	32.80	6.30	0.692	0.499	0.364	-0.680	1.398
BDI exit	3.23	3.14	16.40	10.97	2.643	0.054	2.151	0.860	3.398
STAI State Anxiety baseline	49.23	7.52	54.40	5.77	1.379	0.187	0.726	-0.346	1.776
STAI Trait Anxiety baseline	52.62	7.96	59.00	4.53	2.131	0.053	0.880	-0.208	1.942
STAI State Anxiety exit	37.15	9.703	44.2	5.975	1.501	0.153	0.790	-0.288	1.845
STAI Trait Anxiety exit	36.31	9.621	51.4	10.383	2.921	**0.010**	**1.537**	**0.360**	**2.677**
Low Self Esteem (EDI-3) baseline	12.85	5.16	18.80	3.42	2.364	**0.031**	**1.244**	**0.111**	**2.344**
Personal Alienation (EDI-3) baseline	14.85	8.01	17.00	2.83	0.578	0.571	0.304	-0.737	1.336
Interpersonal Insecurity (EDI-3) baseline	13.54	7.85	13.80	6.14	0.067	0.948	0.035	-0.997	1.066
Interpersonal Alienation (EDI-3) baseline	12.85	5.70	11.00	4.53	-0.646	0.527	-0.340	-1.373	0.703
Interoceptive Deficits (EDI-3) baseline	17.00	8.35	14.40	3.51	-0.664	0.516	-0.350	-1.383	0.694
Emotional Dysregulation (EDI-3) baseline	8.62	7.63	6.80	3.90	-0.501	0.623	-0.263	-1.295	0.776
Perfectionism (EDI-3) baseline	16.92	4.55	18.40	3.51	0.650	0.525	0.342	-0.701	1.375
Ascetism (EDI-3) baseline	13.15	5.41	15.40	5.50	0.785	0.444	0.413	-0.634	1.448
Maturity Fears (EDI-3) baseline	15.46	7.29	19.80	10.38	1.009	0.328	0.531	-0.524	1.570
Low Self Esteem (EDI-3) exit	3.85	3.41	16.40	5.37	5.977	**0.00002**	**3.145**	**1.628**	**4.615**
Personal Alienation (EDI-3) exit	7.38	6.16	11.20	4.92	1.235	0.235	0.650	-0.415	1.695
Interpersonal Insecurity (EDI-3) exit	7.62	5.78	13.60	4.45	2.076	0.054	1.092	-0.020	2.175
Interpersonal Alienation (EDI-3) exit	7.77	4.68	9.60	4.28	0.760	0.458	0.400	-0.647	1.434
Interoceptive Deficits (EDI-3) exit	8.69	7.18	14.20	4.92	1.565	0.137	0.824	-0.258	1.881
Emotional Dysregulation (EDI-3) exit	4.77	5.12	5.60	6.27	0.291	0.775	0.153	-0.882	1.183
Perfectionism (EDI-3) exit	13.54	5.22	16.80	5.26	1.185	0.253	0.623	-0.439	1.667
Ascetism (EDI-3) exit	8.00	5.07	11.00	6.20	1.061	0.304	0.558	-0.499	1.599
Maturity Fears (EDI-3) exit	8.38	6.45	16.20	10.78	1.913	0.074	1.007	-0.095	2.081

Supplemental Table 2. Comparison successful versus non-successful completers eating disorder behaviors and trait variables (two-sided t-test)

	EDE-Q Global < 2 (n=13)			EDE-Q Global > 2 (n=5)			t	p	d	95% Confidence Interval	
	M		SD	M		SD				Lower	Upper
**EDE Global past month baseline**	4.02	±	0.76	4.35	±	0.78	0.838	0.415	0.441	-0.608	1.477
**EDE-Q Restraint past month baseline**	3.32	±	1.12	3.20	±	1.65	-0.184	0.857	-0.097	-1.127	0.937
**EDE-Q Eating Concern past month baseline**	3.22	±	1.35	3.36	±	1.00	0.216	0.832	0.113	-0.920	1.144
**EDE-Q Shape Concern past month baseline**	4.94	±	0.63	5.55	±	0.52	1.900	0.076	1.000	-0.101	2.073
**EDE-Q Weight Concern past month baseline**	4.06	±	0.85	4.76	±	0.78	1.593	0.131	0.838	-0.244	1.897
**EDE Global past month exit**	0.95	±	0.56	3.73	±	0.95	7.786	**0.0000008**	**4.097**	**2.331**	**5.819**
**EDE-Q Restraint past month exit**	0.50	±	0.50	2.88	±	1.44	5.375	**0.020**	**2.829**	**1.388**	**4.222**
**EDE-Q EatingConcern past month exit**	0.51	±	0.43	2.65	±	0.96	6.683	**0.006**	**3.517**	**1.905**	**5.082**
**EDE-Q ShapeConcern past month exit**	1.46	±	0.83	4.66	±	1.33	6.195	**0.00001**	**3.260**	**1.714**	**4.759**
**EDE-Q WeightConcern past month exit**	0.96	±	0.62	4.28	±	1.21	7.759	**0.0000008**	**4.083**	**2.320**	**5.801**
**EDI3-DT baseline**	19.38	±	5.72	25.00	±	2.45	2.090	0.053	1.100	-0.014	2.183
**EDI3-B baseline**	7.69	±	6.59	5.20	±	6.38	-0.725	0.479	-0.381	-1.415	0.664
**EDI3-BD baseline**	27.77	±	7.62	32.60	±	7.60	1.206	0.245	0.634	-0.429	1.679
**EDI3-DT exit**	10.08	±	7.06	22.60	±	4.83	3.618	**0.002**	**1.904**	**0.661**	**3.104**
**EDI3-B exit**	1.92	±	2.81	3.20	±	3.90	0.778	0.448	0.409	-0.638	1.444
**EDI3-BD exit**	13.62	±	7.24	32.40	±	7.96	4.807	**0.0002**	**2.530**	**1.158**	**3.855**

Supplemental Figure 5. Correlation graphs for EDI-3 Low Self-Esteem at exit and EDE-Q Global. Restraint, Eaing, Shape and Weight Concern, and EDI-3 Drive for Thinness

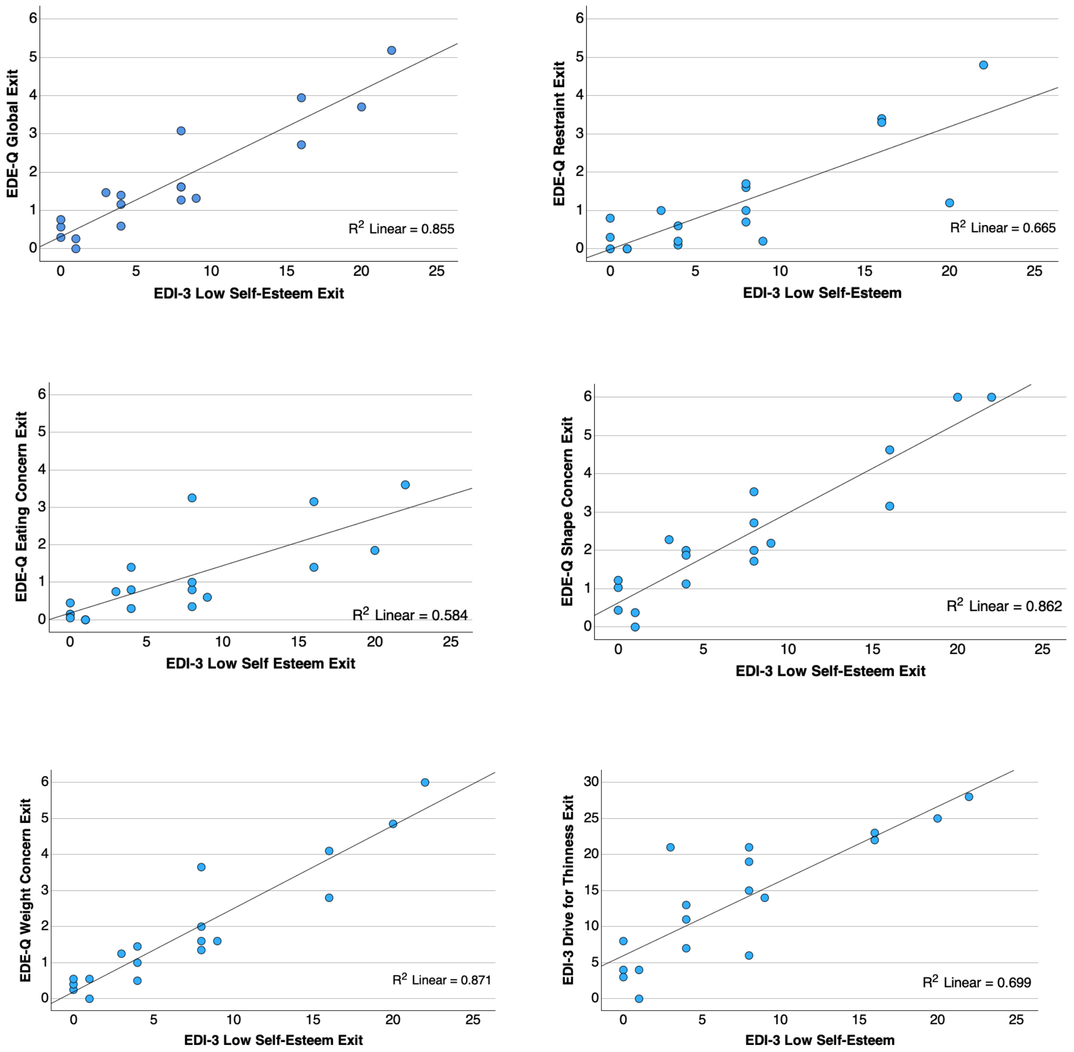

Supplemental Figure 6. Correlation graphs for weekly ketone levels (beta hydroxybutyrate, BHB) and EDE-Q Global scores

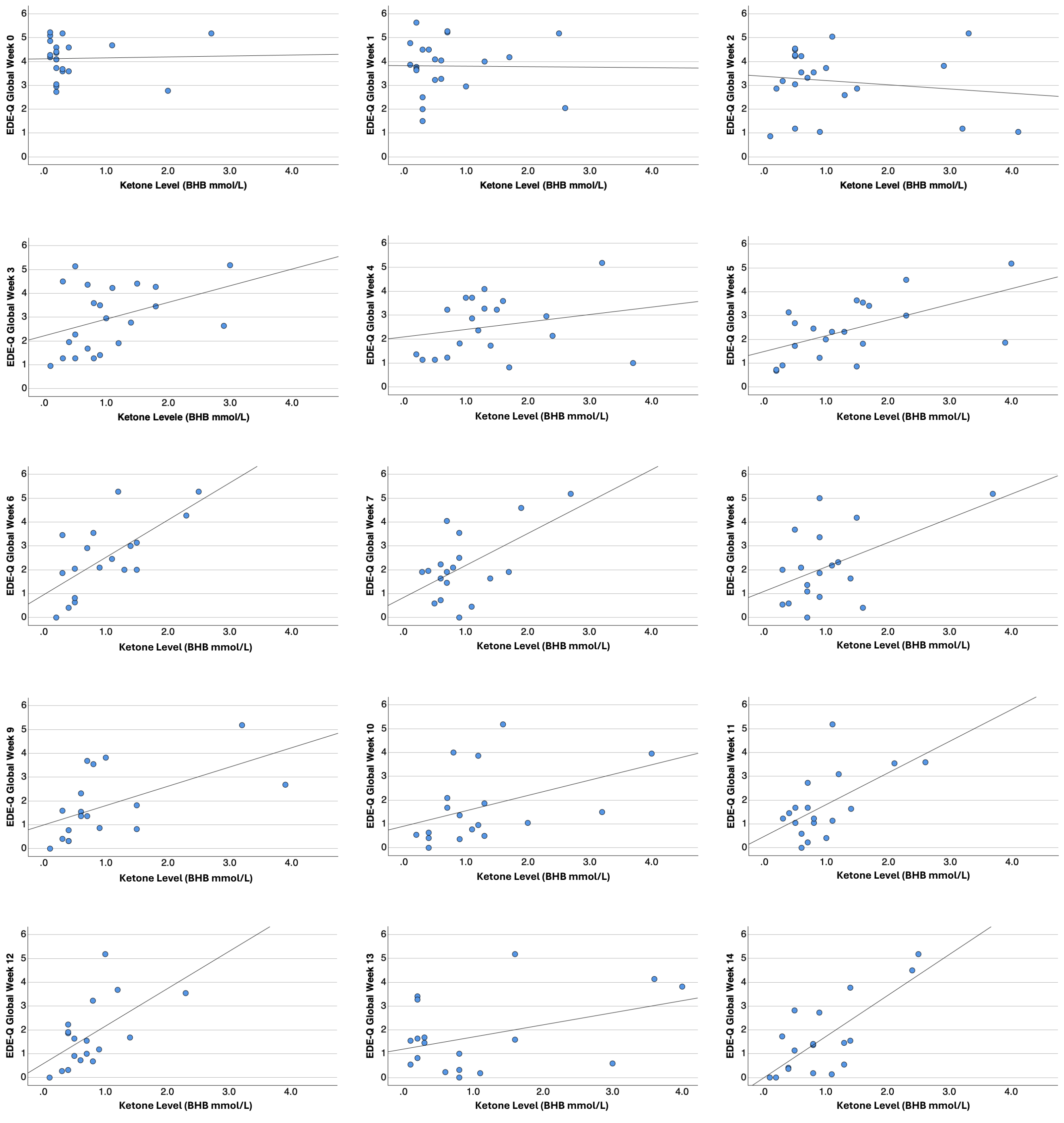

Supplemental Figure 7. Correlation graphs for weekly ketone levels (beta hydroxybutyrate, BHB) and trust in hunger and satiety cues scores

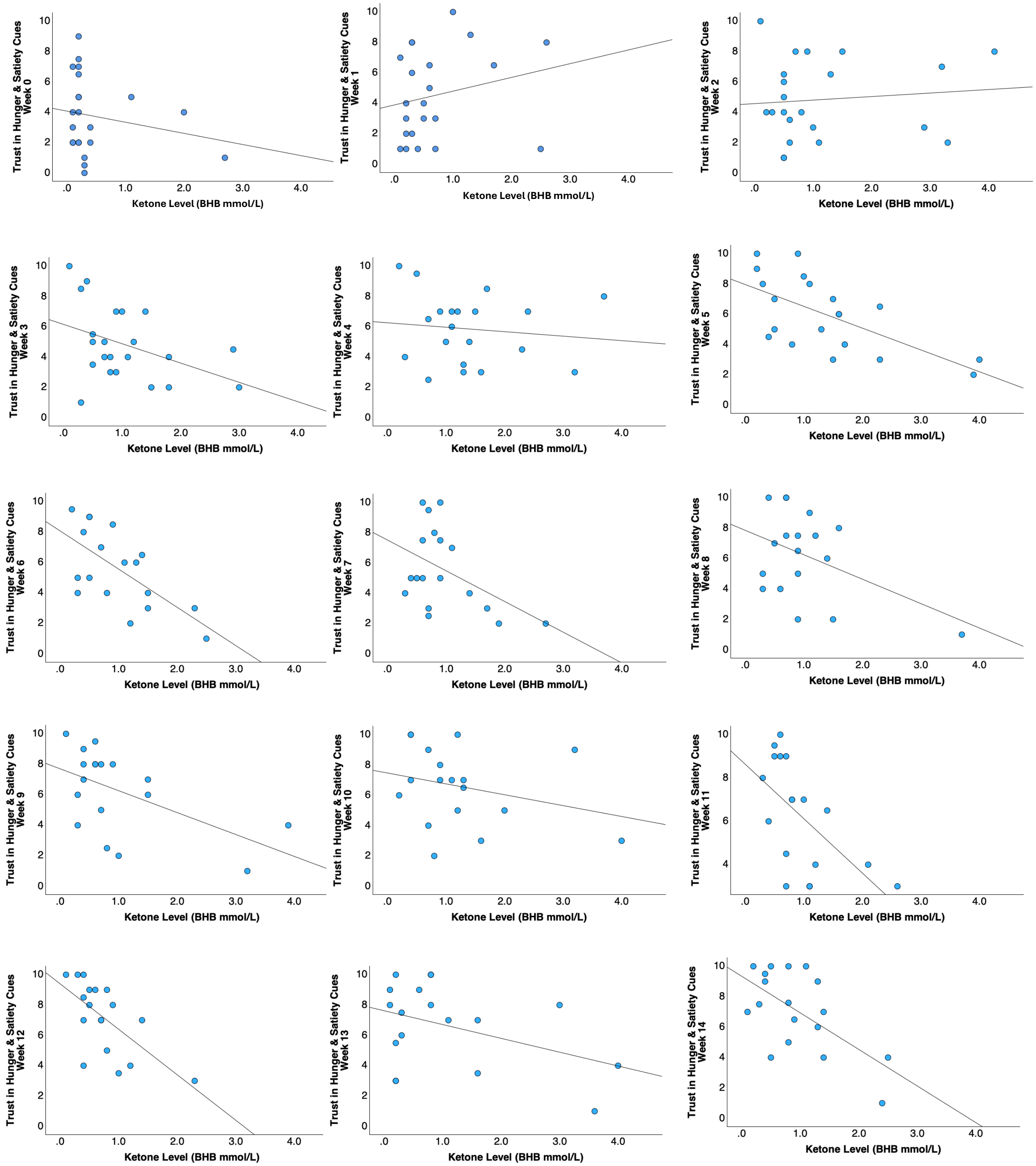

Supplemental Figure 8. Correlation graphs for weekly ketone levels (beta hydroxybutyrate, BHB) and BDI depression scores

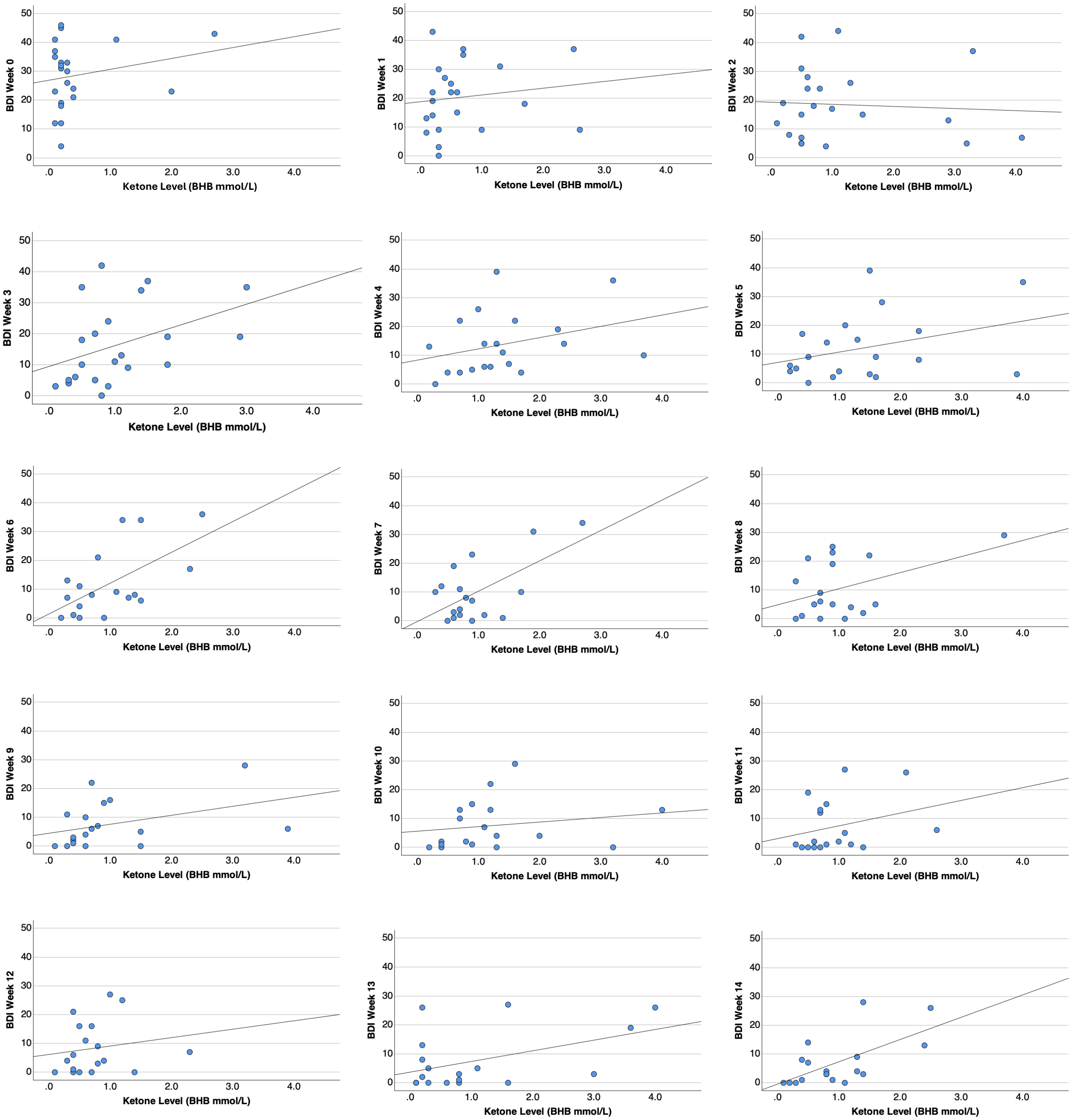

Supplement: Supplementary file 1 — Supplemental Material [file 43856_2026_1644_MOESM1_ESM.pdf]
